# Linking tumor with systemic environment in the search for novel biomarkers in breast cancer

**DOI:** 10.1186/2051-1426-3-S2-P87

**Published:** 2015-11-04

**Authors:** Sotirios P Fortis, Louisa G Mahaira, Michael Sofopoulos, Nectaria Sotiriadou, Christoforos Haritos, John F Voutsas, Nikolaos Anastasopoulos, Niki Arnogiannaki, Christopher Shipp, Graham Pawelec, Sonia A Perez, Constantin Baxevanis

**Affiliations:** 1 Cancer Immunology and Immunotherapy Center, Saint Savas Cancer Hospital, Athens, Greece; 2 Pathology Department, Saint Savas Cancer Hospital, Athens, Greece; 3 Tuebingen University Hospital, Tuebingen, Germany

## Background

Recent studies linking immune signatures in cancer with prognosis have provided unequivocal proof of the essential role of the immune system in tumor pathophysiology. They have also initiated a quest for immune biomarkers that could serve as intermediate endpoints of response to therapy and outcome in cancer. In view of growing evidence for interplay between tumor and systemic circulation, it is critically important to define relationships between the tumor and the periphery in order to reveal novel prognostic and/or predictive biomarkers for cancer.

## Methods

Herein, we correlated density and localization of tumor infiltrating immune cells with levels of circulating cytokines/chemokines and mi-RNAs in breast cancer (BCa) patients with invasive ductal carcinoma without neoadjuvant therapy. To this end, fifty BCa patients were enrolled with written informed consent. Serum was obtained from patients one day before surgery and compared with normal donors. The analysis of immune cell infiltrates (CD4+, CD8+, FoxP3+ and CD163+) was performed by IHC in paraffin-embedded tissues. The density (i.e. the number of positive cells per mm^2^ tissue surface area) and the relative ratios among the different subpopulations, in the tumor center and the invasive front, were analyzed. As indicators of peripheral immunity several cytokines/chemokines (i.e. ΤGF-β1, ΙL-1Ra, IL-9, IL-10, RANTES) were determined in patient serum using LUMINEX^®^ platform technology. The levels of circulating mi-RNAs (miR-21, miR-23a, miR-146a) were estimated by qRT-PCR.

## Results

Significant correlations among cytokines/chemokines or mi-RNAs in sera were detected among patients but not in healthy donors. There were also correlations between several cytokines/chemokines and/or mi-RNAs with the density and location of different immune-cell subpopulations intra-tumorally. Importantly, we also established correlations between the relative distribution of different immune cell subpopulations within the tumor and specific circulating factors. Thus, we observed a direct correlation between circulating RANTES and CD4/CD8 ratio in the tumor center, whereas at the same time RANTES negatively correlated with the CD4/CD163 ratio at the invasive front.

## Conclusions

Our results show for the first time that the differential distribution of immune-cell subpopulations in the tumor microenvironment as well as their ratios are associated with a functional indication in the periphery which is reflected in a significant relationship with different systemic immune profiles. We believe that these local and systemic immune signatures either alone or in combination may represent more sensitive biomarkers for the clinical outcome of BCa patients. However, their prognostic and/or predictive significance remains to be evaluated in the clinical setting.

